# Letter from the Editor in Chief

**DOI:** 10.19102/icrm.2018.091009

**Published:** 2018-10-15

**Authors:** Moussa Mansour


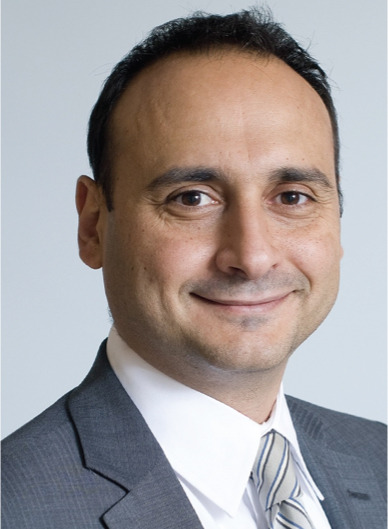


Dear Readers,

As atrial fibrillation (AF) affects a large number of patients and results in a major clinical and economical burden, research has focused on, among other things, the elucidation of risk factors for AF such as obesity, sleep apnea, hypertension, and the excessive use of alcohol in an attempt to better understand the condition.^1,2^ Surprisingly, there has also been a growing focus on high-intensity physical training as a risk factor for the development of AF. For many years, physical training was thought to be beneficial in that it improved longevity and promoted physical and mental health. Prior epidemiological research has demonstrated a J-curve relationship between the intensity of physical training and the risk of developing AF.^3^ However, the cause of such a relationship has not been sufficiently determined to date. As an example, one hypothesis is that heavy exercise can promote inflammation, which in turn leads to AF; still, the exact mechanisms of such an association remain poorly understood, and studies aimed at clarifying this relationship have been limited to preclinical animal trials.

Recently, important research was presented at the 2018 European Society of Cardiology in Munich, Germany. Despite the inclusion of only a small number of patients, it demonstrated that high-level endurance sports athletes have more atrial fibrosis in comparison with individuals in a control group. The study,^4^ by Dr. David Peritz and colleagues in Utah, used magnetic resonance imaging to detect AF. As such, this study offers an important mechanistic explanation regarding the association between AF and the level of physical training in humans.

This is a significant development for many of us who see a large number of patients with AF and who try to educate them about lifestyle modifications to control this disease. While talking about losing weight, controlling sleep apnea, and reducing the intake of exercise alcohol is relatively well-accepted, discussing the relationship between exercise intensity and AF is counterintuitive to many patients. The findings of this study and hopefully others conducted in the future will provide us with the information needed to better discuss this subject with patients. It is important to note, though, that many questions remain unanswered, including the impact of gender in athletes on the development of AF and regarding the optimal amount of exercise that results in a maximum health benefit without an increase in the risk of AF.

Best wishes for a healthy and productive fall season.

Sincerely,


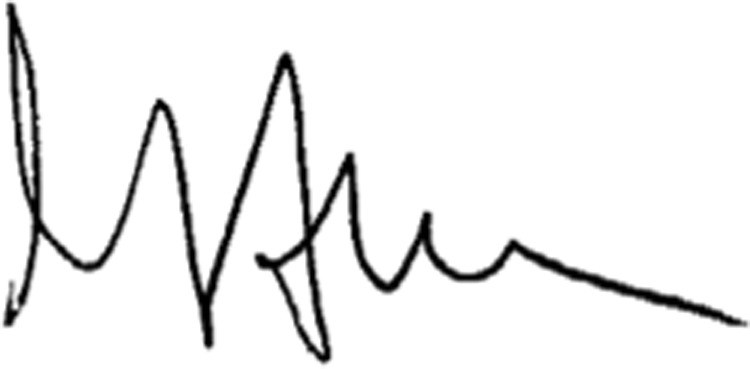


Moussa Mansour, md, fhrs, facc

Editor in Chief

The Journal of Innovations in Cardiac Rhythm Management

MMansour@InnovationsInCRM.com

Director, Atrial Fibrillation Program

Jeremy Ruskin and Dan Starks Endowed Chair in Cardiology

Massachusetts General Hospital

Boston, MA 02114
